# Near-Infrared Femtosecond Laser Ablation of Au-Coated Ni: Effect of Organic Fluids and Water on Crater Morphology, Ablation Efficiency and Hydrodynamic Properties of NiAu Nanoparticles

**DOI:** 10.3390/ma14195544

**Published:** 2021-09-24

**Authors:** Niusha Lasemi, Günther Rupprechter, Gerhard Liedl, Dominik Eder

**Affiliations:** 1 Institute of Materials Chemistry, Technische Universität Wien, 1060 Wien, Austria; guenther.rupprechter@tuwien.ac.at (G.R.); dominik.eder@tuwien.ac.at (D.E.); 2 Institute of Production Engineering and Photonic Technologies, Technische Universität Wien, 1060 Wien, Austria; gerhard.liedl@tuwien.ac.at

**Keywords:** femtosecond laser processing, crater morphology, depth profiling, NiAu NPs, multiangle dynamic light scattering, nanofluids, scanning electron microscopy, micro X-ray diffraction

## Abstract

Scanning electron microscopy (SEM) and profilometry of the crater morphology and ablation efficiency upon femtosecond laser ablation of Au-coated Ni targets in various fluids revealed a pronounced dependence on the ablation medium. For ethanol, a sufficient ablation efficiency was obtained, whereas for 2-butanol a higher efficiency indicated stronger laser–target interaction. Hierarchical features in the crater periphery pointed to asymmetrical energy deposition or a residual effect of the Coulomb-explosion-initiating ablation. Significant beam deviation in 2-butanol caused maximum multiple scattering at the crater bottom. The highest values of microstrain and increased grain size, obtained from Williamson–Hall plots, indicated the superposition of mechanical stress, defect formation and propagation of fatigue cracks in the crater circumference. For n-hexane, deposition of frozen droplets in the outer crater region suggested a femtosecond-laser-induced phase explosion. A maximum ablation depth occurred in water, likely due to its high cooling efficiency. Grazing incidence micro X-ray diffraction (GIXRD) of the used target showed residual carbon and partial surface oxidation. The produced nanoparticle colloids were examined by multiangle dynamic light scattering (DLS), employing larger scattering angles for higher sensitivity toward smaller nanoparticles. The smallest nanoparticles were obtained in 2-butanol and ethanol. In n-hexane, floating carbon flakes originated from femtosecond-laser-induced solvent decomposition.

## 1. Introduction

Nanoparticle (NP) generation by laser ablation in liquids was first reported in 1987 [1]. Improvements in laser parameters allowed control of the size, morphology and composition of laser-synthesized nanoparticles [2,3,4], opening a variety of applications in medicine [5,6,7], photonics [8], electronics [9], energy conversion [10] and catalysis [11,12]. During pulsed laser ablation in liquid (PLAL), several processes (e.g., ionization of matter, plasma evolution, cavitation creation, bubble expansion and collapse, nanoparticle formation and secondary processes) occur sequentially or simultaneously [13,14]. The interaction of a femtosecond laser beam with a target results in local material vaporization and the formation of a high-temperature plasma plum. Since the molten layer of the target and the plasma plum are in direct contact with the liquid, a supercritical temperature liquid can be formed that assists the formation of a short-lived and hemispherical membrane, a so-called cavitation bubble [14], in which primary and secondary nanoparticles are generated and encapsulated [15]. After the collapse of the cavitation bubble, the nanoparticles remain dispersed in solution. However, agglomeration, particle diffusion and ripening processes occur after the cavitation collapse [16].

Ni-based alloy/core-shell nanoparticles have been well-explored in heterogeneous catalysis due to their “cost-effective” properties ([11] and references therein). They also have shown magneto-plasmonic behavior and have potential biomedical applications (e.g., for magnetic resonance imaging) [17].

Nickel–molybdenum nanoparticles were generated by nanosecond laser ablation in water and acetone: in water, the oxidation of particles led to phase segregation, while a homogenous bimetallic distribution was observed in acetone [18]. Moreover, nanosecond laser ablation of Ni foil in an aqueous colloidal silica nanoparticle solution avoided aggregation [19]. Bimetallic nickel–cobalt (Co-rich) nanoalloys were produced by nanosecond laser ablation of Ni_50_Co_50_ in organic liquids and stabilized by N-vinyl-2-pyrrolidone (PVP) [20]. 

Regarding the NiAu alloy NPs, they are specifically interesting as catalysts in steam reforming because a low quantity of single Au atoms in contact with low-coordinated Ni surface atoms already improves the carbon coking resistance tolerance [21,22]. In the previous work, NiAu bimetallic nanoparticles were produced by femtosecond laser ablation in organic fluids and were characterized by means of electron microscopy techniques (high-resolution imaging, electron diffraction and elemental mapping) [11,23]. 

Studies of the ablation properties of targets and the crater morphology in liquids can be employed to determine the ablation efficiency and thus nanoparticle productivity. This requires a systematic study of laser–matter interactions, considering the physicochemical properties of the target material and characteristics of the particular fluid [24,25,26]. 

For ultrashort laser radiation, a high ablation efficiency is expected due to a negligible heat-affected zone (HAZ), high-stress confinement during phase explosion [27] and the absence of plasma interactions with photons (e.g., shielding and scattering) [28]. 

The advantage of using femtosecond systems at low repetition rates <200 kHz is to avoid heat accumulation during the multi-pulse ablation process [29], specifically for the scalable synthesis of nanoparticles in liquids [30]. Furthermore, Niso et al. [31] studied the effect of multi-pulse ablation and incubation behavior on the decrease in threshold fluence. They reported that an increase in laser pulse number (for *N* ≤ 1000) can enhance the material removal via localization of defects in the lattice before saturation. Multi-pulse ablation and crater morphology of steel and copper in long and short pulse regimes were investigated by Chichkov et al. [32]. Femtosecond laser processing by low numbers of pulses resulted in surface structures, whereas higher numbers of shots led to debris-free craters. Holes drilled with nanosecond pulses induced damage and debris due to melting and heat accumulation.

Ultrashort laser ablation also initiates fast processes such as fast plasma cooling and solidification of molten droplets. The plasma temperature for femtosecond pulses is between 5000 and 7000 K [33] with cooling rates in the order of (10^12^ K s^−1^) [14], which is 10^2^ times faster than the cooling rates in the nanosecond regime [34]. 

The appearance of femtosecond-laser-induced surface morphologies such as nanohillocks, voids, pores, bumps and jets was attributed to the Coulomb explosion (CE). The CE process is frequently described for dielectrics and semiconductors [35], but for metals only few theoretical and experimental reports have proposed the Coulomb explosion [36,37,38]. Specifically, the femtosecond pulse interaction with electrons in metals leads to the formation of high-temperature electrons (overcoming the work function and leading to thermionic emission), to the distribution of residual charges in the metallic target and thus to electric field creation [39,40]. The CE process can take place if the created electric field strength is beyond the CE energy threshold. Strong repulsive forces on the charged lattice regions then result in lattice explosion and disintegration [37]. The effect of CE on the irradiated area can be observed by applying a few pulses, since multi-pulse laser interaction transforms the CE condition to phase explosion and crater formation.

High-intensity beam interaction with transparent liquids results in an optical breakdown once the density of electrons in the conduction band exceeds its critical values of 10^18^ to 10^20^ cm^−3^ [41]. Typically, a number of fluctuating bubbles are formed due to the optical breakdown. The bubble dynamics (e.g., rebound and collapse) create a high pressure of ~10 GPa at the liquid/solid interface that leads to nonlinear acoustic energy (e.g., shockwave) induction [42,43,44]. Shockwave emission can create high-pressure and high-temperature conditions at the liquid/plasma interface near the bulk of the material; thus, the ablation environment turns into an active chemical reaction site [45]. 

In a previous study on the multi-pulse incubation behaviour of Ni in butanol, laser-induced solvent pyrolysis and carbon deposition around/in the craters was proposed as a reason for increasing the laser absorptivity, decreasing the ablation threshold and consequently increasing the incubation effect [24,25]. Among multiple factors in ultrafast processing, the ablation environment significantly affects the observables. The chemical composition of the liquid not only affects the ablation efficiency [46,47] but also affects the chemical composition of either targets or NPs, since laser interaction with liquids can lead to solvent photolysis and pyrolysis [23,47,48,49,50]. However, only a few studies on materials in liquids exist which describe the influence of the liquid environment on multi-pulse femtosecond ablation efficiency and crater morphology [46,47,51,52,53,54,55,56,57], so this aspect calls for further experimental investigations. 

The presented study thus deals with multi-pulse irradiation of a Au-coated Ni target in various fluids by a near-infrared femtosecond laser (30 fs, 1 kHz, 800 nm), in order to evaluate the ablation efficiency and the ablation crater morphology. Since ethanol and water are the most commonly used solvents for PLAL, they were included in this study. Moreover, solvents such as 2-butanol or n-hexane with higher carbon content were studied, as carbon deposits may modify the laser/target interaction. To evaluate the ablation process of the NiAu target in various media, scanning electron microscopy (SEM) and optical microscopy were performed after ablation to characterize resulting surface features such as cones, cracks, pores and nanodroplets in the circumference of ablated regions. Profilometry with three-dimensional (3D) map scanning of craters was carried out to quantify the ablation efficiency of NiAu in various fluids. Grazing incidence micro X-ray diffraction (GIXRD) was used to determine the chemical composition, crystallite size and microstrain in the target. Multi-angle dynamic light scattering (DLS) was employed to determine the hydrodynamic characteristics of the laser-synthesized NiAu nanofluids.

## 2. Materials and Methods

### 2.1. Materials 

A nickel target, i.e., polycrystalline foil of ~0.5 mm thickness and 10 × 15 mm^2^ in size (purity ≥99.9%), was purchased from Alfa Aesar (Kandel, Germany). A gold wire (purity ~99.95%) was used for physical vapour deposition (PVD) on the nickel surface. Time-of-flight secondary ion mass spectrometry (ToF-SIMS^5^, IONTOF Münster, Germany) and profilometry (DektakXT stylus, BRUKER, Karlsruhe, Germany) were used to determine a gold thickness of ~50 nm. Only a thin Au film was used so that Ni-rich nanoparticles would be produced (as Au segregates to the surface anyway). Pure solvents (ethanol, 2-butanol and n-hexane; p.a. 99.5%) were provided by Sigma-Aldrich, Vienna, Austria. The different solvents were additionally purified by a polypropene filter (pore size of 0.45 µm; VWR, Vienna, Austria) to remove any particulate matter.

### 2.2. Femtosecond Laser Setup 

A near-infrared femtosecond laser system at a wavelength of 800 nm was used to irradiate the NiAu target. A laser energy of *E* = 250 µJ was used to ablate the targets in water, ethanol, 2-butanol and n-hexane resulting in laser fluences of 6.76 J·cm^−2^, 7.68 J·cm^−2^, 3.58 J·cm^−2^ and 18.02 J·cm^−2^, correspondingly.

**Figure 1:**
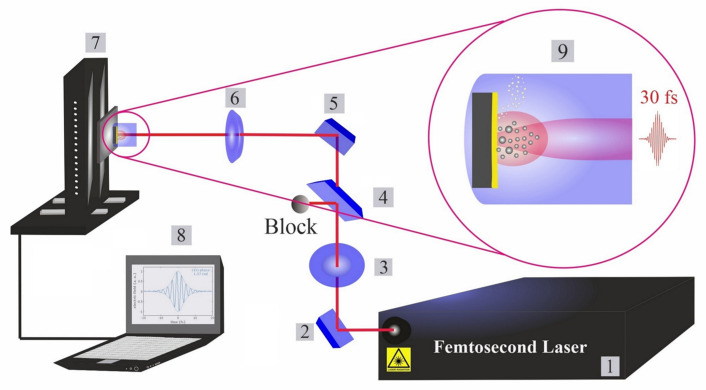
Femtosecond laser ablation and synthesis setup. (1): Femtosecond laser; (2): mirror; (3): half waveplate and polarizer; (4): beam splitter; (5): reflecting mirrors; (6): focusing mirror; (7): motorized XYZ scanning stage; (8): bCNC controller program; and (9): illustration of phases in laser ablation synthesis of nanoparticles in liquids (cavitation bubble growth and the formation of up-flow vapour bubbles).

The femtosecond titanium–sapphire (Ti:sapphire) system operates at a pulse duration of 30 fs, a power of 1 watt and a repetition rate of 1 kHz. A commercial femtosecond system (SPECTRA-PHYSICS, Vienna, Austria, 800 nm, 75 MHz, ≤400 mW) was used as a seeding laser. A chirped pulse amplification (CPA) technique was performed to amplify the system. A Nd:YAG laser (@532 nm) is used to pump the system for extra amplification. The structure of the femtosecond apparatus has been explained in a previous piece of research [23]. An illustration of the typical experimental setup is presented in Figure 1. The beam intensity is controlled by a variable attenuator with a beam splitter and polarizer (THORLABS, Newton, NJ, USA), and is measured by a power meter (Ophir Photonics).

To focus the beam on a target, a parabolic mirror with a focal length of *f* = 101.6 mm is employed. The focal position in various fluids is experimentally determined by ablating a silicon target at different distances from the parabolic mirror. Ablated areas are measured by optical microscopy (Zeiss AxioVision software, Vienna, Austria). The NiAu target was placed inside a cylindrical glassy reactor (liquid thickness of ~15 mm) with an optical window for the horizontal beam entry to avoid optical instability. Furthermore, when compared to the typical vertical beam path configuration [2]), the current experimental setup with horizontal beam path is beneficial for improving the synthesis efficiency, because gravity decreases secondary processes such as beam–NP interactions, especially in the case of long-pulse-duration lasers (as NPs move out of the beam). The laser processing was performed by a motorized XYZ scanning stage and moved by a bCNC command. The optical images of the craters formed by PLAL in various fluids were taken by light microscopy (OLYMPUS-GX51, Vienna, Austria), evaluated by dedicated software (OLYMPUS Stream Motion 2.4, Vienna, Austria). Since a Gaussian beam distribution is assumed; the squared diameter of the ablated area (*D*^2^) is assessed by optical microscopy, enabling the Gaussian beam radius (*w*_0_) to be deduced [58]. The correlation of laser energy (*E*) and fluence (*F*) are defined by Equation (1) [58].

(1) $$F=\frac{2E}{\pi w_{0}{}^{2}}$$

Ultrashort pulse broadening in liquids normally occurs for pulses ≤100 fs [59]. The calculated final pulse durations Δt_out_ in various fluids (Table 1) go beyond the original pulse duration of the system (30 fs). However, they remain in the femtosecond range. The liquid thickness is assumed to be 15 mm.

A Gaussian beam profile of the femtosecond laser affects the crater shape and, accordingly, ablated craters with a conical shape were detected. To calculate the volume of craters, an ideal cone was assumed: ($V=\pi r^{2}h/3$), where V is the ablation volume, *r* is the crater radius and *h* is the depth of the ablated crater. The top diameter and depth of a crater were determined by optical microscopy and stylus profilometry, respectively, to make values of crater volumes more accurate. More precisely, the average of measurements from 7 to 10 craters were considered, extracting X and Y profiles from the map scanning process.

### 2.3. Scanning Electron Microscopy (SEM)

Scanning electron microscopy, imaging the craters and surroundings, was performed on a JCM-6000 Versatile Benchtop SEM (5 to 15 kV; Tokyo, Japan). Backscattered electrons (BE) and secondary electrons (SE) operated at 10 kV were used for imaging the overall topology and surface structure, respectively.

### 2.4. Profilometry

To determine ablation properties, a DektakXT stylus profiler (Bruker, Karlsruhe, Germany) with a stylus force of 3 mg was used for surface analysis (via roughness and thickness measurements), depth analysis and 3D morphology. For improved 3D mapping, a resolution of 10 µm/trace was applied. A conically shaped Bruker diamond tip with a bottom radius of 2 µm and an angle of 45° was used to achieve better resolution and accuracy, limiting artefacts in contact profilometry.

### 2.5. Grazing Incidence Micro X-ray Diffraction (GIXRD)

Phase and crystallinity analyses of material around femtosecond-laser-ablated craters on the NiAu target were performed by an XRD system (Empyrean, PANALYTICAL; Cu-Kα radiation (1.5418740 Å), 45 kV and 40 mA). The target was placed on a computer-controlled xyz stage and the desired area was selected by a charge-coupled device (CCD) camera. Then, micro-XRD was performed as grazing-incidence diffraction (GID) to evaluate the ablation craters. The angle of beam incidence, omega (ω) was fixed at 5° for high surface sensitivity. Microbeam focusing was done by using a micro-nozzle to create a narrow rectangular beam with a width of 300 µm and a length of 6 mm. A parallel beam X-ray mirror collimator for Cu radiation was used to decrease the noise in measurements due to the roughness of the examined area, so that defocusing effects can be avoided. The GaliPIX^3D^ detector (Malvern PANalytical, Malvern, UK) position was varied from 20° to 100° to obtain the XRD pattern.

### 2.6. Dynamic Light Scattering (DLS) 

**Figure 2:**
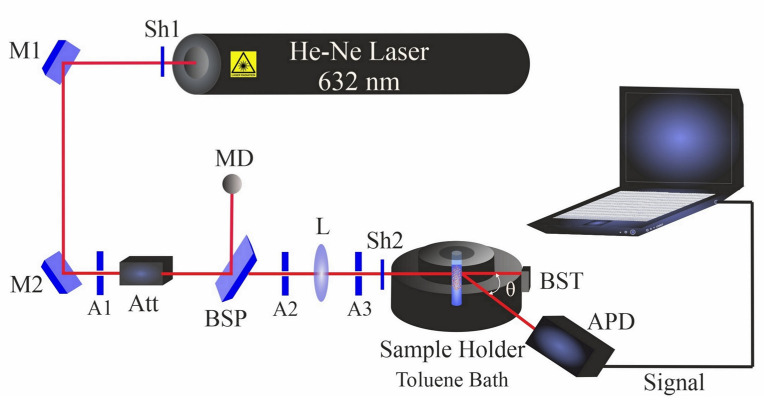
Dynamic light scattering setup. Shutters (Sh1 and Sh2); mirrors (M1 and M2); apertures (A1–A3); beam splitter plate (BSP); attenuator (Att); beam stopper (BST); bispherical lens (L); monitor diode (MD); and avalanche photodiode (APD).

Multi-angle dynamic light scattering (DLS) was carried out using a compact goniometer system (ALV/CGS-3, Hessen, Germany). The DLS system (Figure 2) contains a helium–neon laser that operates at 632.8 nm with a power of 22 mW. Before measurements, colloidal NiAu nanoparticles were ultrasonicated for 5 min in the respective liquid (filtered prior to PLAL to avoid dust particles).

All DLS measurements of NiAu colloidal samples were performed at room temperature with multiple goniometer angles of 60°, 90° and 120°. The angular position of a single detector was changed for angle-dependent data acquisition. 

The hydrodynamic measurements of samples were evaluated by studying the correlation function of the intensity fluctuations. The regularized fit [60] is used to determine the Stokes radius. The polydispersity indices (PDI) were calculated by using a cumulant fit. Γ is the inverse of the mean relaxation time, *D*_t_ is the translational diffusion coefficient and *q* is the scattering wave vector [61]:

Γ = *D*_t_ × *q*^2^ (2)

The scattering wave vector *q* correlates with the refractive index *n*_0_, the wavelength λ and θ is the scattering angle at the position of the detector [62].

(3) $$q=\frac{4\pi n_{0}}{\lambda }\sin\left(\frac{\theta }{2}\right)$$

## 3. Results and Discussion 

Femtosecond laser drilling of micron-sized holes on the Au-coated Ni target was investigated in various fluid media for the same parametric conditions. All experiments were performed at the focal position without applying to defocus. Since ultrashort pulses are short enough not to have any direct interaction with the plasma plume, energy losses due to plasma shielding and plasma scattering can be neglected. The energy of ~250 µJ was used to achieve the maximum ablation rate, based on former studies of ultrashort ablation of Ag [63] and Au [64] targets. Moreover, targets were irradiated by 1000 pulses in various fluids to explore a realistic ablation process for nanoparticle synthesis and to study the effect of accumulative pulses on the target.

### 3.1. Crater Analysis

**Figure 3:**
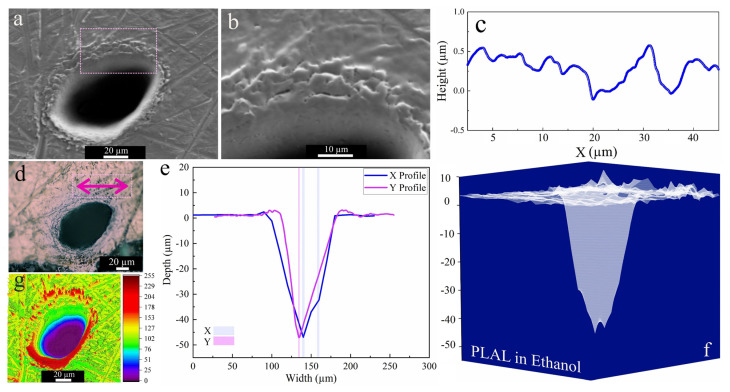
Morphology of a crater formed by PLAL of Au-coated Ni in ethanol (*N* = 1000, 1 kHz, *E* = 250 μJ). (**a**,**b**) SEM image and magnified crater edge; (**c**) a surface profile curve close to the rim; (**d**) optical microscopy of the crater (the green arrow indicates the scan of roughness); (**e**,**f**) depth profile analysis of femtosecond laser-drilled holes and 3D mapping plot; and (**g**) 3D reconstructed image of an SEM image.

Figure 3 shows a characteristic crater after PLAL in ethanol. An average rim height of 2.2 µm was measured for the edge of craters, created by the resolidification of molten material. This material originates from the effect of recoil pressure following the ejection of molten droplets that are favorably stacked in the rim area. Furthermore, nonlinear effects of femtosecond pulse interaction with transparent media may trigger continuum generation, which may be a further reason for surface modification around the rim or outside the crater [65]. The SEM image of the crater perimeter reveals a surface roughness which seems restricted to the upper part of the crater close to the rim. The profilometer indicated an (average) roughness (peak height) of 451 nm, whereas around the rim the surface is generally rather smooth.

Three-dimensional mapping of craters in Figure 3f revealed multiple drilled holes. This indicates multiple internal reflections [66] at the bottom of the ablated craters and/or laser deviation due to interaction with ejected primary and secondary NiAu NPs created during ablation. Along these, the crater edges of femtosecond-processed NiAu in ethanol were smoother (and with almost no HAZ-related damage) than that of nanosecond laser ablation of Ni [25].

**Figure 4:**
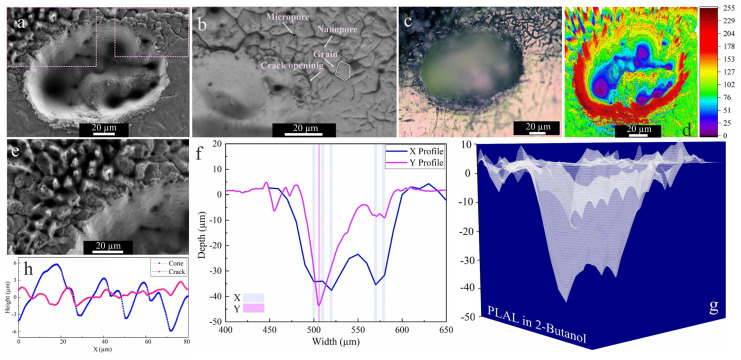
Morphology of a crater formed by PLAL of Au-coated Ni in 2-butanol (*N* = 1000, 1 kHz, *E* = 250 μJ). (**a**,**b**,**e**) SEM images and magnified views of surface morphologies and crater edges; (**c**) optical microscopy of the crater (the green arrow indicates the scan of roughness); (**d**) 3D reconstructed image of an SEM image; (**f**,**g**) depth profile analyses of femtosecond laser-drilled holes and 3D mapping plot; and (**h**) a surface profile curve of the cone and crack areas.

The SEM images of craters obtained by ablation in 2-butanol showed two specific surface morphologies: hierarchical cones and cracks (Figure 4a,b,e). An average surface roughness of 2.18 µm and 1.35 µm was determined for cones and cracks, respectively. This is similar to femtosecond laser ablation of a SiO_2_/Si target in 2-butanol, which had shown similar structures on the top of the craters [47]. Since a horizontal beam delivery was used herein, the beam deviation towards the top of the craters was more pronounced.

The collapse of upward flow of vapor bubbles containing synthesized nanoparticles may be an additional reason for creating defect sites or features surrounding the upper half of the rim. It was reported that inhomogeneous laser energy deposition on a surface containing defects may contribute to the formation of self-organized microstructures (e.g., cones) [67].

These defect zones act as active sites that influence laser energy distribution. Self-organized hierarchical features or spikes (Figure 4e) can grow on these active sites due to consecutively applied laser pulses. Moreover, such conical features or nanohillocks generally develop due to CE processes for ultrashort pulses ≤100 fs [56]. Furthermore, analogous spike features were also detected upon femtosecond laser ablation of a Au target with a single or hundred pulses in air and isopropanol [68].

Similar to ethanol, SEM and 3D mapping of craters ablated in 2-butanol (Figure 4e,f) show several drilled holes. Since the ablation rate in 2-butanol is higher than in the other fluids (see below), a beam deviation due to NPs is more probable and can induce multiple-beam scattering. An average rim height of 2.94 µm was determined for craters after 2-butanol PLAL. In addition to the conical features, microcracks with high-density nanopores and low-density micropores were detected at the outer region of the craters via backscattered SEM imaging (cf. Figure 4b). The average length of microcracks is ~6 µm, with microcrack propagation deflected at grain boundaries at an average angle of 38°. The measured average roughness on hierarchical cones and cracks is 2.18 µm and 1.35 µm, respectively.

The crack formation can be related to the fatigue effect on the target, inducing mechanical load cycles due to consecutively applied pulses (*N* = 1000) on a surface in butanol. Since microcrack propagation in the periphery of craters only occurred in 2-butanol, the fatigue crack dynamics seem solvent-dependent. The dynamic viscosity of 2-butanol is almost three times higher than that of the other liquids, thus the higher vapour pressure at the bubble wall in higher dynamic viscosity fluids [69] may lead to a mechanical effect due to the erosive power of cavitation bubble collapse. Generally, the pressure of bubble collapse is between 1 and 6 GPa [42]. Furthermore, porosity is the most abundant defect feature formed, resulting from entrapped/discharged gases. These micropores may act as local stress enhancers and promote crack nucleation [70,71]. Then, the formation of high-density nanopores is more pronounced and may trigger crack initiation.

**Figure 5:**
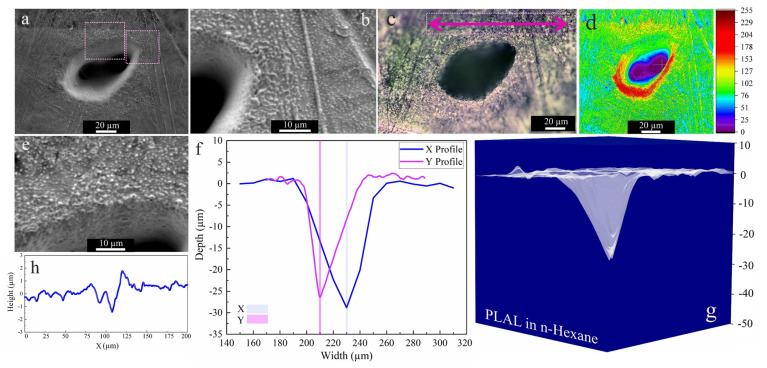
Morphology of a crater formed by PLAL of Au-coated Ni in n-hexane (*N* = 1000, 1 kHz, *E* = 250 μJ). (**a**,**b**,**e**) SEM images and magnified areas of redeposited frozen droplets and crater edges; (**c**) optical microscopy of the crater (the green arrow indicates the scan of roughness); (**d**) 3D reconstructed image of an SEM image; (**f**,**g**) depth profile analyses of femtosecond-laser-drilled holes and 3D mapping plot; and (**h**) a surface profile curve close to the crater.

As the higher carbon number of 2-butanol led to higher ablation efficiency than ethanol, n-hexane was used next. Figure 5 shows an SEM image of an area that was laser-ablated in n-hexane; an average rim height of 1.9 µm is measured after PLAL in n-hexane. Close to the rim, a roughness of 707 nm was observed. Frozen droplets deposited around the craters can be attributed to the femtosecond-induced stress confinement and phase explosion [14,72]. This may be followed by the expulsion of material splashing from the ablation center, with redeposition of particles in the periphery or inside the craters. Nevertheless, only a small ablation efficiency was observed.

**Figure 6:**
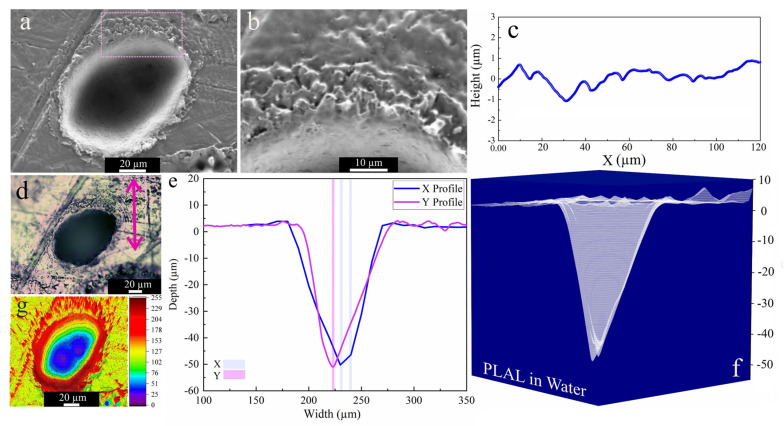
Morphology of a crater formed by PLAL of Au-coated Ni in water (*N* = 1000, 1 kHz, *E* = 250 μJ). (**a**,**b**) SEM image with a magnified area of crater edge; (**c**) a surface profile curve close to the rim; (**d**) optical microscopy of the crater (the green arrow indicates the scan of roughness); (**e**,**f**) depth profile analyses of femtosecond-laser-drilled holes and 3D mapping plot; and (**g**) 3D reconstructed image of an SEM image.

SEM images acquired after PLAL in water (Figure 6b), showed a roughened (~428 nm) area close to the rim, as well as multiple drilled holes analogous to those in ethanol and 2-butanol. An average rim height of ~3.5 µm was measured. For PLAL in n-hexane, the smallest crater diameter, depth, ablation volume and thus specific ablation rate were observed. This finding is similar to that of femtosecond laser ablation of SiO_2_/Si in n-hexane [47]. It may thus correlate with pyrolysis and photolysis of high-carbon-number solvents and the formation of carbon products that impact the ablation efficiency, since ablation of Fe [46] targets in toluene was also low.

As mentioned, upon formation of supercritical plasma the direct contact of plasma and the liquid phase causes a transformation of liquid into the supercritical temperature state [14], which may initiate thermal solvent dissociation. For longer hydrocarbons, the ablation is more chaotic as several chemical products can be expected. For example, solvent oxidation may be catalyzed by freshly synthesized NiAu nanoparticles or the high-intensity beam may lead to photodecomposition of the liquid.

Solvent decomposition then affects the chemical composition of the ablated bulk and the synthesized nanoparticles. High-resolution transmission electron microscopy (HRTEM, Hillsboro, OR, USA) of NiAu NPs femtosecond-laser-synthesized in 2-butanol indicated two types of nanoparticles: with and without a graphite shell [11]. Ni-rich NPs underwent graphitization since Ni is an active catalyst for carbonization, whereas (partially) Au-coated NPs had no signs of graphitization [21]. Apparently, alloying Ni NPs even with a small number of Au atoms suppresses the formation of a graphite shell.

**Figure 7:**
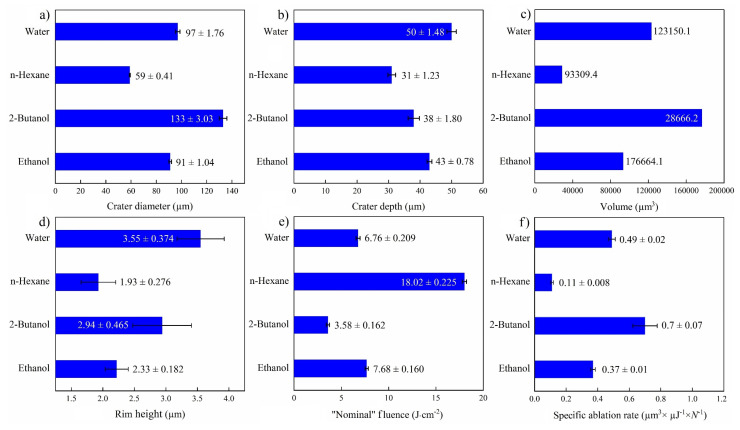
Summary of femtosecond laser ablation properties of Au/Ni in various fluids (*N* = 1000, 1 kHz, *E* = 250 μJ). (**a**) Crater diameter; (**b**) crater depth; (**c**) crater volume; (**d**) rim height, (**e**) “nominal” laser fluence; and (**f**) specific ablation rate.

An overall comparison of ablation properties of femtosecond-laser-drilled Au-coated Ni for various fluids is presented in Figure 7. Former studies on femtosecond laser processing of Fe [46] and SiO_2_/Si [47] reported the smallest spot size in toluene and n-hexane, correspondingly, while using other fluids led to an increase in the ablation threshold and laser fluence. The highest specific ablation rate and ablation volume were obtained in 2-butanol, analogous to a previous study [47]. This may correlate with the deposition of carbonaceous products in the crater, increasing the laser absorption on the surface [24,25]. This decreases the ablation threshold and results in higher productivity. Apart from the physicochemical characteristics of the liquids (e.g., refractive index), the energy deposition on the targets may also be affected by multi-filamentation processes, which is why “nominal” laser fluences are indicated.

The highest ablation depth of the NiAu target was observed for PLAL in water (Figure 7a), similar to femtosecond laser processing of CuZn alloy (brass), with a higher ablation depth in water than in ethanol and air [54]. Femtosecond laser ablation of Fe in water also showed the highest ablation depth in contrast to alcohols, acetone and toluene [46]. A larger ablation depth was reported for water than for alcohol, not only for metallic targets but also for semiconducting targets (e.g., Si) [52]. This can be attributed to the thermal conductivity of water (being higher than that of the other fluids), which leads to a higher cooling rate and reduced thermal effects.

### 3.2. Micro-GIXRD

**Figure 8:**
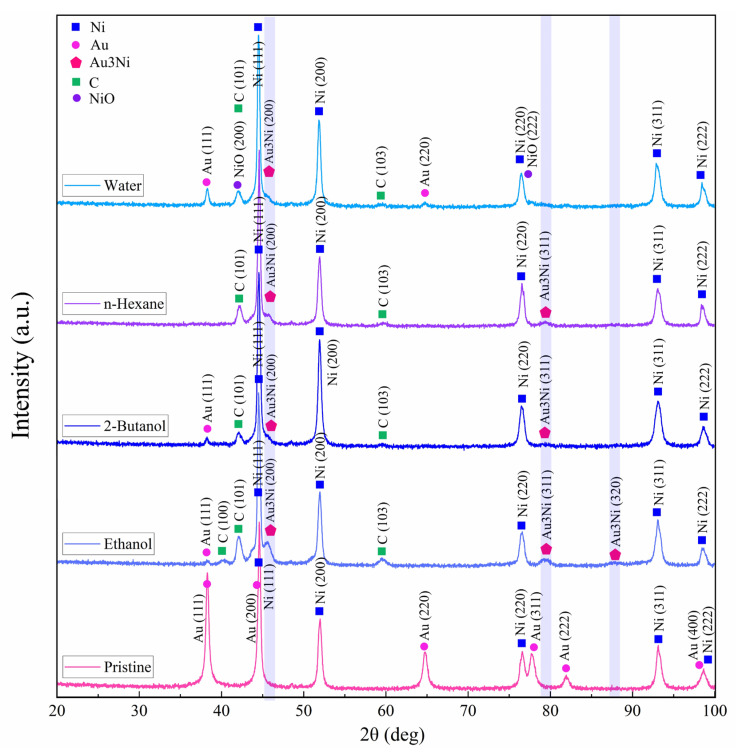
Micro-GIXRD patterns of pristine and femtosecond-processed Au/Ni areas, (*N* = 1000, 1 kHz, *E* = 250 μJ).

XRD diffraction patterns of the target were acquired by micro-GIXRD, both for non-irradiated and femtosecond-laser-ablated zones, with results for various fluids presented in Figure 8. Crystallinity was preserved after ablation. Pristine areas showed intense diffraction peaks related to cubic Ni (JCPDS card no. 01-070-0989) and cubic Au (JCPDS card no. 04-003-5615). Since the target was coated with just a thin film of Au, the Au layer was mostly consumed during ablation.

All laser-treated areas exhibited a tiny peak at 45.3°. This hump detected on the Ni(111) peak may indicate the formation of a Au_3_Ni alloy (JCPDS card no. 04-016-1987) in low concentration, with characteristic peaks at 45.3°, 79.3° and 87.9°. A bimetallic NiAu alloy phase is expected to form above >1083 K [73], when Ni and Au are miscible (note that the plasma temperature achieved by ultrashort pulses is 5 to 7 times higher). After PLAL, especially in water, peaks at 42.2° and 77.1° may be related to the formation of cubic NiO (JCPDS card no. 04-023-3539) due to femtosecond-laser-induced water splitting [74] on Au-coated Ni. The small XRD peaks observed at 42.2°, 42.4° and 59.7° may correspond to anorthic (triclinic) carbon (JCPDS card no. 01-082-9929) [75], in line with probable target carbonization in the case of PLAL in carbon-containing solvents. However, no peaks characteristic of graphite were detected [76], so a full graphitization of the craters upon femtosecond laser irradiation can be excluded.

In a further step, the Williamson–Hall [77] (W–H) method was used to evaluate the microstrain (ɛ) and crystallite size (d_WH_) around the zones ablated in various fluids. The slope and intercept of linearly fitting the W–H plot correspond to the microstrain and crystallite size, respectively. The corresponding Equation (4) is used to determine ɛ and d_WH_:

(4) $$B\cos\theta =\frac{K\lambda }{d_{\mathrm{WH}}}+\varepsilon 4\sin\theta$$

where *B* is the total peak broadening, *K* is a shape factor, *λ* is the X-ray wavelength and θ is the Bragg angle.

**Figure 9:**
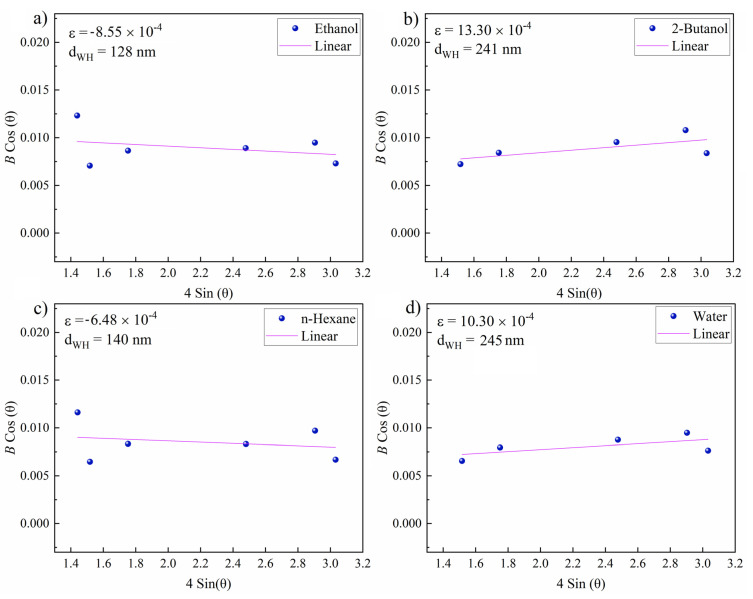
Williamson–Hall plots of femtosecond-laser-ablated areas near craters on the NiAu sample (*N* = 1000, 1 kHz, *E* = 250 μJ). Resulting microstrain (ɛ) and grain size (d_WH_**)** for NiAu ablated in (**a**) ethanol; (**b**) 2-butanol; (**c**) n-hexane; and (**d**) water.

Representative W–H plots are shown in Figure 9. The highest resulting microstrain was measured for 2-butanol, which can be correlated with lattice strain and subsequent peak broadening due to laser-induced fatigue that triggers the formation of defects (e.g., microcracks) or the formation of hierarchical structures (Figure 4) during the occurrence of CE. The large crystallite size of 241 nm can be attributed to the existence of hierarchical cones, pores and cracks. A large crystallite size was also measured for water (245 nm). The lowest (compressive) lattice strain was measured for n-hexane. Thus, a high impact of laser-induced stress on targets seems not to occur in n-hexane. A crystallite size of 148 nm was measured that is close to the size of the expelled spherical particles (Figure 5b,e), deposited close to the crater rim.

### 3.3. Multiangle Dynamic Light Scattering

Dynamic light scattering (DLS) was performed to analyze the hydrodynamic characteristics of nanofluids containing the synthesized NPs. Upon DLS measurements, laser light interacts with nanoparticles in solution and induces random thermal movements that led to a collision with solvent molecules, thus a so-called Brownian motion can occur. The Stokes–Einstein model [78] is used to analyze the Stokes radius (R_s_) and translational diffusion D_t_ coefficient. The translational friction (*f*_trans_) [79] is also considered since NP motion in solutions can apply friction that is directly related to R_s_ and liquid viscosity.

Theoretical studies of the formation of metal nanoparticles by ultrashort pulses suggested a bimodal size distribution including primary (~10–20 nm) and secondary nanoparticles (~50–60 nm) [14,80]. However, the DLS (at a specific single scattering) angle yields a mean value that may be an average from two or more values. Thus, it is difficult to extract the contributions of different particle sizes in the colloidal solution. Typically, the size difference between different modes should be by a magnitude of three times in order to be able to observe a second mode at a single angle detection.

Multiangle DLS for three different scattering angles was thus applied to increase the sensitivity for the detection of smaller nanoparticles and to decrease measurement noise. Elastic Rayleigh scattering occurs for particles that are smaller than λ/10 and inelastic Mie scattering is probable for NPs that are larger than that [79]. Thus, light scattering at the forwarding direction dominates for particles that are larger than the wavelength of the light, whereas smaller NPs scatter light in almost all directions. Therefore, backscatter detection reduces unwanted contributions of dust particles, whereas scattered light from smaller NPs can be efficiently monitored. Accordingly, apart from scattering angles of 60° and 90°, a scattering angle of 120° was applied to better evaluate smaller particle sizes and to mask the larger ones. Smaller nanoparticles diffuse faster than larger ones, and thus autocorrelation curves at various angles show diverse decays. A faster decay is thus expected at larger scattering angles, corresponding to smaller nanoparticles.

**Figure 10:**
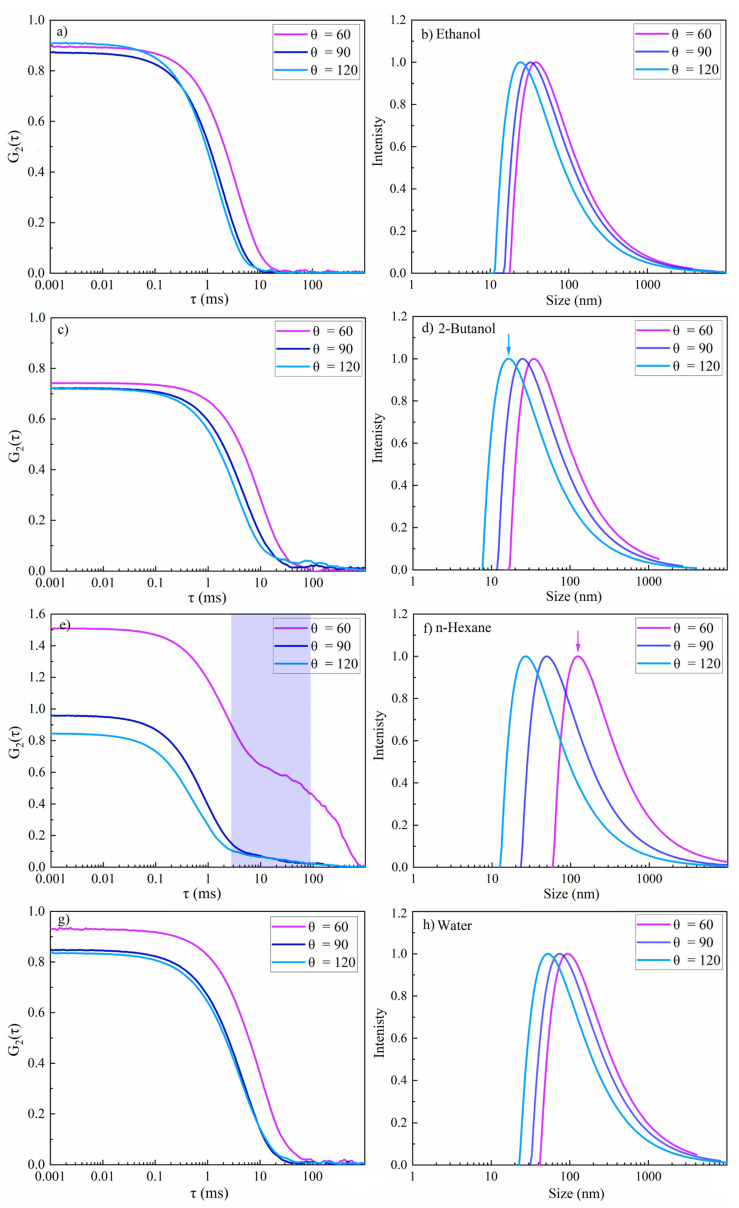
Multi-angle dynamic light scattering of femtosecond-laser-synthesized colloidal nanoparticles in various fluids (*N* = 1000, 1 kHz, *E* = 250 μJ). (**a**,**c**,**e**,**g**) The autocorrelation function of the intensity versus delay time (τ) and (**b**,**d**,**f**,**h**) the intensity-weighted size distribution results. (**a**,**b**) Ethanol; (**c**,**d**) 2-butanol; (**e**,**f**) n-hexane; and (**g**,**h**) water.

The results of multiangle DLS of colloidal NiAu NPs in various fluids are presented in Figure 10, including the intensity-weighted size distribution, and are also summarized in Table 2. The intensity autocorrelation function G_2_(τ) curves for NPs in ethanol, 2-butanol and water exhibited a smooth exponential decay. Good-quality data are confirmed as the maximum of the autocorrelation intensity is near 1, as expected. In contrast, the G_2_(τ) curve for n-hexane at θ = 60° showed a more complex decay, similar to femtosecond-laser-synthesized Si NPs [47], which indicates carbon by-products [48]. The scattering angle of 60° is not satisfactory for NiAu NPs in n-hexane, because the amplitude of the correlation function is larger than 1. Nevertheless, the second decay related to multimodality was only detected at this angle. The second decay at τ = 10 ms (Figure 10e) is thus related to coarse particles (not detected at 90° and 120°). This demonstrates the increased sensitivity for smaller particles at larger angles. The resulting R_s_ values at different DLS angles are also shown in Table 2. The smallest R_s_ was obtained for 2-butanol (marked by the blue arrow in Figure 10b), similar to a previous study [47]. This may suggest less particle agglomeration due to the formation of a few atomic layers of graphite shell, which may act as a capping agent, or due to a steric hindrance of agglomeration by floating amorphous carbon flakes [11]. The largest hydrodynamic radius (marked by the pink arrow in Figure 10f) and polydispersity index (PDI) were observed for n-hexane (Table 2), evident from a second decay that corresponds to larger particles. In contrast, for ethanol, 2-butanol and water, a moderate polydispersity (~0.1–0.4) was observed.

The hydrodynamic radius was measured at 120° for NiAu NPs in water and n-hexane was 113 nm and 59 nm, respectively. Similarly, based on TEM and AFM analysis of femtosecond-laser-synthesized Ni NPs [81], Arboleda et al. reported larger NPs in water (TEM ~8–21 nm; AFM ~2–26 nm) due to Ni oxidation than in n-heptane (TEM ~4–8 nm; AFM ~2–7 nm). Furthermore, primary and secondary nanosecond laser ablation of Ni in organic solvents led to lognormal size distribution and also exhibited a dependency to the specific heat value of solvents. In n-hexane, due to its lower specific heat than that of water, a lower amount of ablated material was observed. Vice versa, in water the amount of ablated material was high which, together with the formation of nickel oxide shells around crystalline Ni cores, lead to larger NP sizes [82].

## 4. Conclusions

In PLAL, employing the non-equilibrium nature of femtosecond laser pulses, the type of fluid can be used to affect the ablation morphology and efficiency. Scanning electron microscopy of the ablated target, which is Au-coated Ni foil, revealed two particular surface morphologies: hierarchical cones and fatigue cracks. The hierarchical cones can nucleate on active sites (e.g., defect zones) as these sites cause non-uniform laser energy deposition, so applied consecutive laser pulses can assist in the growth of conical features on the surface.

Microcracks and pores were formed at the periphery of the craters upon PLAL in 2-butanol. These fatigue cracks may be viscosity-dependent and can be formed by a mechanical effect due to bubble collapse. GIXRD peak broadening resulting from defect formation indicated the highest microstrain and a large grain size formed in 2-butanol. The formation of rims is related to the resolidification of materials molten during the ablation process, especially in water. Several frozen droplets surrounding the craters upon PLAL in n-hexane point to a femtosecond-laser-induced phase explosion, resulting in molten material ejection from the center of the ablated craters. The evolution of multiple holes at the bottom of craters is related to multiple light scattering during laser ablation in liquids.

Concerning the specific ablation rate of a femtosecond-laser-processed NiAu target in various liquid media, an order of 2-butanol > water > ethanol > n-hexane was observed. The lowest ablation rate in n-hexane may be due to laser absorption by floating carbonaceous by-products, resulting in significant energy loss. Chemical composition analysis of the target surface after PLAL by micro-GIXRD revealed the presence of Au_3_Ni, NiO and C in the irradiated areas.

The nature of the liquid also affects the resulting particle sizes of the produced colloids. Multiangle dynamic light scattering of colloidal NPs, especially at larger angles, allowed the characterization of smaller nanoparticles with higher sensitivity. The lowest Stokes radius was observed for NPs produced in 2-butanol. This may be due to the formation of a thin graphite shell around the nanoparticles that prevents particles from further diffusion and growth. The largest nanoparticle sizes were obtained by PLAL in water, which can be attributed to an increased aggregation or formation of hydroxide shells around the nanoparticles. Complex multiple decays in the autocorrelation curve for NPs prepared in n-hexane, specifically at a 60° scattering angle, may be due to the presence of floating carbon flakes in the colloidal system, originating from femtosecond-laser-induced solvent dissociation. The presented results should help in the selection of suitable conditions targeting high ablation efficiency and desired particle size ranges.

## Figures and Tables

**Table 1:** Calculated final pulse duration (τ_P_) after passing through the liquid.

Medium	Water	Ethanol	2-Butanol	Hexane
τ_P_ (fs)	45	65	96	85

**Table 2:** Summary of evaluated hydrodynamic results of femtosecond-laser-synthesized colloidal nanoparticles.

Solvent	θ (deg.)	*R*_s_ (nm)	PDI	*D*_t_ (µm^2^/s)	*f*_trans_ (kg s^−1^)
Ethanol	120°	53	0.20	3.8	1.07 × 10^−9^
	90°	70	0.16	2.9	1.41 × 10^−9^
	60°	81	0.24	2.5	1.63 × 10^−9^
2-Butanol	120°	36	0.40	1.9	2.10 × 10^−9^
	90°	53	0.37	1.3	3.09 × 10^−9^
	60°	70	0.18	1.0	4.08 × 10^−9^
n-Hexane	120°	59	0.48	13	3.22 × 10^−10^
	90°	109	0.47	7	5.95 × 10^−10^
	60°	265	0.53	3	1.44 × 10^−9^
Water	120°	113	0.45	2	2.17 × 10^−9^
	90°	156	0.36	1.4	2.99 × 10^−9^
	60°	188	0.36	1.1	3.61 × 10^−9^

## Data Availability

Not applicable.
